# In spite of good intentions: patients' perspectives on problematic social support interactions

**DOI:** 10.1186/1477-7525-3-52

**Published:** 2005-09-05

**Authors:** Carla Boutin-Foster

**Affiliations:** 1 Division of General Internal Medicine, Weill Medical College of Cornell University, 525 E. 68 th Street, Box 46- Baker Tower 14, New York, New York 10021, USA

**Keywords:** Qualitative study, Social support, Social networks, Coronary artery disease

## Abstract

**Background:**

In the setting of an acute coronary syndrome, the natural inclination of friends and family members is to provide social support. However, their efforts may be perceived as being problematic or unhelpful. The objective of this study was to identify the characteristics of problematic social support interactions from the perspectives of patients.

**Methods:**

This was a qualitative study among a purposive sample of 59 patients who had been hospitalized for an acute coronary syndrome. Patients were asked: "Can you describe the types of things that your family members, close friends, and health care providers did during this period to try to be helpful or supportive but you felt was unhelpful or felt that it caused you more stress." Responses were analyzed using qualitative techniques and reviewed by two independent corroborators.

**Results:**

The types of behaviors performed by social network members that were perceived as being unhelpful were grouped under 5 themes: (1) excessive telephone contact, (2) high expression of emotions, (3) unsolicited advice, (4) information without means for implementation, and (5) taking over.

**Conclusion:**

Patients in this study described actions of their social network members that were intended to be supportive but instead were perceived as problematic because they were in excess of what was needed, they were incongruous with what was desired, or they contributed to negative feelings. Helping social networks to understand the potential problematic aspects of social support can aid in tailoring effective social support interventions.

## Background

Acute coronary syndromes such as unstable angina or myocardial infarction account for approximately 2.5 million hospitalizations in the United States annually [1,2]. The period surrounding an acute coronary syndrome is often marked by fear, anxiety, and uncertainty about the resumption of activities such as work or sexual activity [3-5]. Under these circumstances, social support is often mobilized as a resource to help patients cope with their illness. Social support is a set of interactive and dynamic processes in which particular actions or behaviors are directed at an individual to positively effect his or her social, psychological, or physical well-being [6]. Social support can be provided in the form of emotional support such as providing love and affection, tangible support that is provided by giving practical assistance with a task, and informational support that is provided by giving guidance or advice [7].

There has been a lot of enthusiasm and interest in the benefits of social support in coronary artery disease. However, this enthusiasm has been tempered by studies demonstrating that too much social support can be problematic. Revenson uses the metaphor of a "double-edged sword" in describing social support interactions [8]. She describes positive and problematic support from social networks as two different domains that can coexist; efforts to provide social support can alleviate stress and can also augment stress.

Much of the work on the problematic aspects of social support interactions has been in the setting of chronic conditions such as arthritis, HIV, and cancer. In a study among patients with rheumatoid arthritis, problematic social support interactions was associated with greater fatigue [9]. Among patients with HIV, unsupportive social interactions correlated with greater depression [10]. Among breast cancer patients, having unsupportive social network interactions was associated with lower emotional well-being and worse social functioning [11]. Many studies have documented the positive aspects of social support among coronary artery disease patients. However, less is known about problematic social support interactions in this setting.

The experience of having an acute coronary syndrome provides a useful context for understanding problematic social support interactions. Because this is a period marked by high emotions and stress, the natural inclination of social networks is to try to be helpful. However, their efforts to provide social support, though well-intended, may be perceived as problematic. The objective of this qualitative study was to identify characteristics of problematic social support interactions between patients who were hospitalized for an acute coronary syndrome and different members of their social networks. The term problematic social support is used to refer to instances of support provided by social networks that were perceived as non-supportive, even though the provider's actions may have been well-intended [8]. The goal is to utilize results from this study to help social networks understand the potential problematic aspects of their intention to provide social support and to enhance the effectiveness of social support interventions.

Although previous studies have provided important information on potential problematic aspects of social interactions, they have had some limitations which, this study will address. One limitation is in the measurement of social support. Most studies have used quantitative instruments to measure unsupportive behavior and have measured problematic social support interactions along a continuum from helpful to unhelpful [12,13]. A quantitative approach to studying negative aspects of social support quantifies the degree of unhelpfulness for the purpose of statistical correlations. However, this method of measurement does not provide detail regarding the specific types of behaviors that are perceived as being unhelpful. Another limitation of previous studies is their focus on one dimension of social support, namely emotional displays of support. This study will build upon earlier findings and expand upon this work by using a qualitative approach to gain greater insight into specific types of behaviors that are perceived as unsupportive. The study also focuses on informational and tangible examples of problematic social support interactions in addition to emotional social support interaction.

## Methods

### Study design and participants

This was a qualitative study conducted as part of a larger prospective study designed to examine the impact of social support interactions on the health outcomes of patients being evaluated for an acute coronary syndrome. The setting was the cardiac telemetry unit of a tertiary care hospital. Participants were recruited using purposive sampling; a technique often used in qualitative research to recruit participants who are best suited to provide answers to the question of interest [14]. As opposed to statistical sampling, purposive sampling involves the deliberate choice of respondents, and is concerned with how well a sample represents a population of interest. This is done by identifying a group of patients with the particular experience or condition of interest. In this study, the population of interest was patients who had experienced an acute coronary event and who were able to describe interactions with social networks.

### Data collection

Interviews were conducted using a semi-structured open-ended questionnaire. The interviewer followed the participant's train of thought while making sure to cover the desired topic. Patients were asked to reflect on the period surrounding a prior hospitalization for an acute coronary syndrome. Participants were then given the following probe: "When people are hospitalized for a heart condition, their family members, close friends, and health care providers often try to be helpful." Patients were then asked: "Can you describe the types of things that your family members, close friends, and health care providers did during this period to try to be helpful or supportive but you felt was unhelpful or felt that it caused you more stress?" Because health care providers have been cited as providers of social support in previous studies, they were included as potential social networks [15]. Most of the interviews occurred within 24 to 48 hours of admission. Therefore, patients were not asked about the current hospitalizations since it is possible that it would be too soon to allow for sufficient interactions with social networks. Recruitment continued until data saturation. Data saturation is the term used in qualitative research to describe the point at which responses become redundant and additional recruitment does not yield new responses [16]. The duration of the interviews was approximately 30 minutes. Interviews were conducted in the hospital and at the patient's bedside. Interviews were not audio-taped because the presence of other patients and hospital staff in the room created an environment that was often too noisy and therefore not conducive for audio-taping. There was also a concern that other patients or staff in the room would be recorded without their knowledge and consent.

### Data analysis

A fundamental goal of qualitative analysis is to identify central themes that represent a particular phenomena or experience. In this study the goal was to identify problematic social support interactions between patients and social networks in the context of an acute coronary artery event. Data was analyzed line-by-line through a series of consecutive steps known as open coding, axial coding, and selective coding. Open coding is an analytic process in which data is "opened-up" or dissected line-by-line to reveal underlying meaning of a particular experience or phenomena. The initial step in open coding involves identifying concepts which are events, incidents, or ideas that are described by the respondent and that relate to a particular phenomenon. Concepts can be in-vivo quotes which, are the exact words used by the respondents or they can be names assigned by the coder based on a particular imagery evoked. In this study in-vivo quotes were used to describe concepts because they best reflect what was said by respondents. Similar concepts were then grouped to form categories which are explanatory terms that represent a group of concepts. Categories can be divided into discrete components or subcategories that further describe the characteristics. In the initial step of this analysis, several abstract concepts or in-vivo terms were selected. In the next series of steps, data was reduced to more discrete components. The next step was axial coding, where the focus was on looking for shared properties between categories and subcategories. Data was reassembled to form more precise and complete explanations of the particular phenomena or question of interest. Finally, selective coding was done to identify central categories or themes that represent main ideas that were being conveyed [16].

In effort to ensure the trustworthiness of the data, several steps were taken [17,18]. First, a wide range of participants who had different experiences and who were from different age groups were recruited to ensure that the findings would be transferable or generalizable. Second, detailed notes of each interview were maintained and reviewed throughout the coding process and weekly meetings were held to refine concepts and categories. When there were discordant views regarding the interpretation of findings, the raw data was reviewed and new categories were derived until a consensus was reached [19,20]. Finally, two independent corroborators who were not part of the initial coding process reviewed the original transcripts and decided whether they agreed with the final concepts and categories. In order to maximize the validity of the findings, the first 30 patients were interviewed in the presence of two interviewers who compared notes after each interviewer [21]. The study methods and protocol were approved by the Institutional Review Board on the Conduct of research.

## Results

Data saturation was achieved at 59 participants. Table 1. describes the demographic and clinical characteristics of study participants. The mean age was 67 years, 42% were female, 24% were African-American, 10% were Latino, and 84% of patients had completed high school. The majority of patients had been transferred to the telemetry unit for further evaluation of unstable angina. The characteristics of interactions of social support that were perceived as being unhelpful or problematic were grouped under 5 themes: (1) excessive telephone contact, (2) high expression of emotions, (3) unsolicited advice, (4) information without means for implementation, and (5) taking over. Examples of these themes and some associated categories and subcategories are shown in table 2.

**Table 1:** Demographic characteristics of study participants

**Demographic characteristics**	**n = 59**
*Age(years) *± *SD*	67 ± 12
*Female*	42%
African-American	24%
Latino-American	10%
Married	56%
Employed	54%
High School graduate	84%
**Clinical characteristics**	
Hypertension history	54%
Diabetes	30%
Unstable Angina	51%
Previous infarction	45%
Previous Heart failure history	15%
Severity of illness (CCS) Class IV*	39%

**Table 2:** Example of coding

**In vivo quotations**	**Categories**	**Sub-categories**	**Themes**
"I don't like it when there are too many phone calls"	Specific behaviors Telephone contact Telephone ringing	Bothersome behaviors Too much contact	Excessive telephone contact
"I know she worries because she cares but she worries too much"	Worries Conflicts Conflicts between worrying and caring	Too much concern Too emotional	High expression of emotions
"I know you want to say something but don't give unsolicited advice"	Constant advice Others always Speaking Conflicts	Unwanted help Conflict between wanting information and needing advice	Unsolicited advice
"Thanks, for the advice but I know I need to adjust my diet but give me the means with which to do it"	Advice Health behavior advice Solutions	Impractical Conflict between wanting information and needing advice	Information without means for implementation
"They treat me like an invalid. I'm an independent person, respect that"	Control Personal treatment Sense of person Self-perception Independence Respect	Lack of control Too much control Lost control	Taking over

### Excessive telephone contact

When reflecting upon previous hospitalizations, several participants described how close family members and friends called them during their hospitalization and upon discharge. In general, participants said that these telephone calls were welcomed. However, when the calls became "too much" they became unhelpful. This theme was endorsed by both male and female participants. Family members and friends were most often cited as social networks who engaged in this type of activity. As an example, in describing the experience during hospitalization, one participant said, *"I don't like it when there are too many phone calls. I hate the phone ringing all of the time"*. Another example came from a participant describing the telephone calls she received at home after a hospitalization for unstable angina. This participant described the calls from her friend as follows, *"They were becoming too demanding. If she called, she expected me to drop what I was doing and talk to her"*.

### High expression of emotions

Participants described the period surrounding the hospitalization as being very emotional for themselves and their social network members. There was a general understanding that their social networks were responding out of genuine concern. Responses that were grouped under this theme often pertained to the reaction of family social networks, especially adult children. One participant said about her daughter: *"I know she worries because she cares but she worries too much"*. Another participant described how her children engaged in arguments because they worried so much about her. She said, *"They worry so much. They argue and fight about who is going to take care of me. I don't like when they fight over me"*. Another participant said, "they *drive me crazy with concern"*. This theme of over expression of emotions is perhaps best articulated in the following response, *"I don't want anyone to pity me, cry over me, or try to search for encouraging words to say. Just be quiet and support me. You can support me without saying anything"*.

### Unsolicited advice, information or assistance

Receiving advice and information on making health behavior changes also emerged as a dominant theme in this population. The consensus was that while the advice was appreciated, it was often given without any solicitation on the participant's part. As one participant said: *"I know you want to say something but don't give unsolicited advice"*. Another participant said, *"People talk and give advice when all I want them to do is listen to me"*. This aspect of receiving advice was also often mentioned in the context of the patient-provider interaction, where patients felt that the provider told them more information than they wanted to hear. One participant said, *"Sometimes doctors tell patients too much"*. Another patient said, *"Don't tell me things that are going to worry me"*.

### Information without practical means for implementation

Conversely, there were participants who stated that they wanted to receive information from their health care providers, however the information was often unaccompanied by specific guidance or a means for practical implementation. *"I really don't like it if someone tells me all the things I should be doing, but doesn't teach me how to do those things"*. Another example was, *"They always tell me that I should do this or that, but it's easy for them to say. It's not their body"*. Another respondent said, *"Thanks, for the advice but I know I need to adjust my diet but give me the means with which to do it"*.

### Taking over

In addition to providing emotional support and advice, social networks also tried to be supportive by providing tangible assistance. However, some participants perceived this assistance as an attempt to "take over" their lives. For example one participant said, *"They want me to move me into a retirement home, but what will happen to my things when I move to the retirement home?" *This participant described how the family was willing to pay for a home and physically move her. Another participant described how his son became his source of transportation but this soon became problematic. He said, *"I like to drive, but my son tells me I can't drive because of my condition. He drives me everywhere"*. Another participant stated *"They treat me like an invalid. I'm an independent person, respect that"*.

## Discussion

Most of the literature on social network interactions describes the more positive aspects of receiving social support. There are few studies on the potential problematic side-effects of social support interactions. After a coronary event, it is common for patients to be fearful, to feel vulnerable, and to have feelings of depression [22-24]. Social support networks often rally around patients and try to help them cope with this stress by providing different forms of social support. However, as described by patients in this study, social networks may unknowingly exacerbate their negative feelings. Instead of alleviating stress, they may contribute to what patients described as feelings of being "over-protected", "being more stress", and "feelings of invalidism". The behaviors of social networks that engendered these feelings were grouped under 5 themes: (1) excessive telephone contact, (2) high expression of emotions, (3) unsolicited advice, (4) information without means for implementation, and (5) taking over.

Interestingly, in describing "negative or problematic" interactions, most patients actually began their story with a positive statement such as "I know they mean well" or "they worry because they care". Therefore, rather than contradicting existing theories on social support, these findings actually expand upon this construct and present a variation or another extreme of social support interactions.

According to social science theories, emotional, informational, or instrumental social support describe the types of psychological and material resources provided by social networks that are intended to benefit an individual's ability to cope with and respond to stressful situations. Social support is thought to exert its positive impact by diminishing psychological or physical stress[25,6,26]. Social support theory also suggests that social support interactions may function along two extremes; interactions that have positive and salutary benefits as well as interactions with negative consequences [8]. The findings of this study reflect the negative extreme of social support interactions. These findings also demonstrate what Helgeson and colleagues described as social controlling aspects of social support. For example the insistence and advice from social networks on modifying health behaviors may be forms of informational support but may be also viewed as efforts to take control or a sense that they are not in control [27].

Qualitative research often uses an inductive or bottom-up approach whereby themes are derived from observations. This was the primary mode of analysis used in this study. However, a unique aspect of this study is that as a secondary validation step, a deductive (top-down approach) was also employed in order to determine whether the themes that were derived made sense in light of existing categories of social support. This step showed that the above themes could be linked to traditionally held categories regarding positives social supports, namely emotional, informational, and tangible support. For example, the themes "excessive telephone contact" and "high expression of emotions" can be interpreted as excessive emotional social support. Instead of alleviating stress, excessive displays of emotion may contribute to excessive worry or guilt in the recipient. The themes "giving unsolicited advice" and "providing information without a means for implementation" can be viewed as describing inadequate or unsatisfactory informational support. Efforts to provide informational support may be perceived as being problematic if it is incongruous with the patient's desire to either seek or avoid information. The theme "taking over" can be interpreted as describing unbridled tangible support. In spite of the benefits of tangible support, efforts to provide tangible support may not be well-received if it contributes to a sense of loss of control or makes the recipient feel more vulnerable [28]. Therefore, instead of diminishing the impact of stress, problematic social support interactions may contribute to an increase in stress or negative feelings.

In extrapolating these findings, there are methodological limitations that need to be addressed. Specifically, with regard to the study design, the strategy used in selecting study participants, and the approach to data analysis. First, the use of a cross-sectional study design limits the ability to follow up patients and determine the impact of these behaviors on health outcome or whether perceptions of social support fluctuate over time. Second, the use of purposive sampling as a strategy to select participants may enhance the internal validity of findings but because it involves non-random sampling, it may also introduce selection bias and limit generalizability [29]. Third, the unit of analysis in this study was the patient's perceptions. The data might have been enriched by also eliciting and analyzing the characteristics of social networks and the relationship such as the duration of the relationship or prior experience of the social network with coronary artery disease. Future studies may wish to elicit the social support provider's point of view in addition to the recipients'. Future studies may also build upon these findings by evaluating the recipient's internal cognitive structures such as coping style and the patient's locus of control or beliefs about who is in control of one's health [30-32]. Other variables such as depression and perceived stress would also provide greater insight into factors that underlie perceptions of helpfulness.

In spite of these limitations, these findings provide guidance for suggesting more effective social network recommendations. During an acute illness, health care providers often function as liaisons between the patient and their social networks. Thus, they are in a unique position to engage social networks and their respective loved ones in a discussions on the types of support that are most helpful and those that are not [21]. Social networks should be encouraged to set realistic goals that balance their needs with that of the recipients. Patients should be encouraged to discuss with their social networks examples of behaviors that are not helpful. Patients should be encouraged to effectively communicate their request of the type of social support, the amount, and timing of support. Social networks must also understand that in spite of their best intentions their efforts to be helpful may be perceived as being unhelpful. Social support is a complex and multifaceted construct, understanding the problematic aspects in addition to the supportive aspects is important to effectively tailoring interventions that utilize social support to improve health.
